# Steroid Premedication Might Protect From Postoperative Erythema Nodosum Leprosum in Leprosy: A Case Report

**DOI:** 10.7759/cureus.55986

**Published:** 2024-03-11

**Authors:** Shweta B Hembrom, Ghazal Ahmed, Habib Md R Karim, Vineeta Singh, Priyanka Rai, Suchita V Meshram

**Affiliations:** 1 Anesthesiology, Critical Care, and Pain Medicine, All Indian Institute of Medical Sciences, Deoghar, Jharkhand, IND; 2 Dermatology, Venereology and Leprosy, All India Institute of Medical Sciences, Deoghar, Jharkhand, IND; 3 Anesthesiology, Critical Care, and Pain Medicine, All India Institute of Medical Sciences, Deoghar, Jharkhand, IND; 4 Obstetrics and Gynecology, All India Institute of Medical Sciences, Deoghar, Jharkhand, IND

**Keywords:** postoperative complicaiton, drug-related side effects and adverse reactions, multi drug therapy, type 2 lepra reaction, immune-mediated inflammatory diseases, hansen’s disease

## Abstract

Leprosy is known for its diverse pathophysiologic involvement and resulting multisystemic manifestation and morbidities. Despite global efforts to eliminate this public health illness, it is still prevalent in some Asian and European countries. Perioperative management of a leprosy patient is challenging owing to the indirect and direct involvement of the airway, respiratory, and cardiac systems; treatment-related side-effects involving the hepato-renal systems affecting the anesthesia techniques and drugs pharmacokinetic and pharmacodynamics. While anaesthesiologists are aware of such happenings and often tailor the anesthesia management for the concerning issues, immunological aspects of the disease and drug-related adverse events are less enquired about, such as type-2 lepra reaction, i.e., erythema nodosum leprosum (ENL), etc. Further, data on perioperative ENL management and prevention are still being determined. We report one case of a 52-year-old female who underwent gynecology surgery and developed ENL on the third postoperative day, which was managed using Steroids. Unfortunately, the patient had a surgical site infection, which required another surgery within the month, while the patient was still under the steroid successfully without any adverse events. Although a single case cannot provide causation or association, the case is presented to highlight the probable preventive action of steroids on the occurrence of postoperative ENL, where surgical stress is considered a risk factor.

## Introduction

Leprosy is caused by an acid-fast bacillus, i.e., *Mycobacterium leprae*, which is well-known and still prevalent in India even in the post-elimination era [1]. As a complication, it affects the skin, mucosa, peripheral nervous, and immunological systems [2]. Although curable with early detection and multidrug therapy (MDT) using Dapsone, Clofazimine, and Rifampicine, if left untreated, the disease may cause progressive and permanent disabilities [2]. However, the drugs used for the treatment have significant adverse effects.

Perioperative management of leprosy patients requires multiple critical aspects to be considered. Due to the disease's multisystemic involvement, especially its involvement in the airway, respiratory, and cardiac systems, a tailored and vigilant management approach is required [3]. Airway management, hemodynamics, postoperative recovery, positioning-related musculoskeletal, and mucosal injury prevention are vital [3]. Moreover, MDT drugs can also affect the hepatic and hematological systems and complicate the perioperative period by altering drug metabolism and excretion along with drug-drug interaction [4].

While leprosy and lepra reaction cases are frequently encountered by dermatologists, as anesthesiologists, encountering these patients for some planned surgical procedure is rare and relatively scarce in the literature today [5]. Nevertheless, evidence of the perioperative lepra reaction and its prevention strategy is either absent or hardly reported. Here, we are presenting a case report of a patient who was diagnosed with Lepromatous Leprosy five months back, on MDT, who underwent combined spinal-epidural anesthesia for total abdominal hysterectomy and postoperatively developed erythema nodosum leprosum (ENL). The patient underwent surgical intervention twice. The second sitting was under steroid coverage, which highlights the importance of steroid pretreatment for possibly preventing perioperative lepra reactions.

## Case presentation

A 52-year-old female presented to our institute with chief complaints of right-sided lower abdominal pain. After clinical examination and ultrasonography, she was diagnosed with right-sided serous cystadenoma. The patient was planned for a total abdominal hysterectomy and bilateral salphingo-oophorectomy. On the pre-anesthesia clinic evaluation, she gave a history of suffering from Leprosy and being under treatment with an MDT-multi bacillary (MDT-MB) regimen for the last four months. She did not have features of neuritis or joint stiffness at that time. She was also allergic to Ciprofloxacin and Ibuprofen.

Laboratory investigations revealed mild anemia, altered albumin globulin ratio, mild hyponatremia, and increased erythrocyte sedimentation and C-reactive protein levels; the rest of the routine investigations were fairly within normal levels (Table 1). Chest x-ray and resting 12-lead electrocardiogram also did not reveal any significant abnormalities.

**Table 1:** Preoperative blood investigation results with reference ranges. SGOT: serum glutamic-oxalacetic transaminase, SGPT: serum glutamic-pyruvic transaminase.

Investigations	Result	Reference value
Haemoglobin	10.2 g/dL	11.5 – 14.5 g/dL
Total Leucocyte count	18.61 thou/mm^3^	4 – 11 thou/mm^3^
Polymorph	77.1%	40% – 75%
Lymphocytes	16.1%	20% – 40%
Monocytes	6.7%	2% – 10%
Eosinophils	0.1%	0% – 6%
Basophils	0.0%	0% – 2%
Platelet count	388 thou/mm^3^	150 – 400 thou/mm^3^
Erythrocyte sedimentation rate	51.5 mm/hr	0 – 19 mm/hour
C-reactive Protein	146.33 mg/L	< 5 mg/L
Prothrombin time	13.8s	11.9 – 15.6s
International Normalized Ratio	0.99	< 1.5
Random Blood Sugar	80 mg/dL	70 – 140 mg/dL
Total Bilirubin	0.70 mg/dL	0.2 – 1.2 mg/dL
Direct Bilirubin	0.20 mg/dL	0 – 0.3 mg/dL
Indirect Bilirubin	0.50 mg/dL	0 – 0.9 mg/dL
SGPT	32 U/L	< 40 U/L
SGOT	48 U/L	< 40 U/L
Total Protein	8.8 g/dL	6.0 – 8.0 g/dL
Albumin	3.6 g/dL	3.7 – 5.3 g/dL
Globulin	5.2 g/dL	2.3 – 3.6 g/dL
Blood urea	23.0 mg/dL	10 – 40 mg/dL
Serum creatinine	0.8 mg/dL	0.5 – 1.2 mg/dL
Serum Sodium (Na^+^)	131.3 mEq/L	136 – 146 mEq/L
Serum Potassium (K^+^)	3.86 mEq/L	3.5 – 5.1 mEq/L
Serum Chloride (Cl^-^)	91.3 mEq/L	101 – 109 mEq/L

Preoperatively, she was advised to take nil per os for eight hours for solid food and two hours for clear fluid. The dermatologist altered her MDT-MB regimen to minus Dapsone, and the rest (Clofazimin and Rifampicine) was continued in the perioperative period. She was also asked to take a tablet of Alprazolam 0.25 mg the night before surgery and a tablet of Pantoprazole 40 mg on the morning of the surgery. Informed consent was obtained for surgery and anesthesia.

Surgery was conducted under combined spinal and epidural (CSE) anesthesia. Applying the American Society of Anesthesiologists (ASA) standard monitoring and co-loading, the CSE technique was performed in a sitting position. An epidural catheter was placed at the level of L2-L3 using the loss of resistance to air technique, followed by a subarachnoid block (SAB) using a 25 G Quincke spinal needle at the level of L3-L4. Bupivacaine heavy 0.5%, 15 mg was administered for SAB. Epidural augmentation was done intraoperatively using Bupivacaine 0.25% 5mL (12.5 mg) after 40 minutes. Except for a brief episode of hypotension (treated using crystalloid bolus and injection of Mephentermine 3+3 mg), the procedure was uneventful. Postoperative analgesia was achieved with paracetamol 1gm intravenous q8h and epidural top-up with Bupivacaine 0.125% and Morphine 5 mg in 8 mL total volume, required two bolus dosages over the next 48h.

On the third postoperative day, some skin eruptions with itching and pain, especially over the back and abdomen, were noted. Dermatology consultation led to the diagnosis of ENL, and management was started with prednisolone 40mg once daily and other supportive therapy. Unfortunately, she also developed a surgical site infection. She received an injection of Ceftriaxone and a Linezolid tablet for postoperative infection, Pantoprazole 40 mg, a tab of extended-release Iron, and Vitamin C 500 mg once a day as supportive management. MDT-MB without Dapsone was continued, and she underwent debridement and secondary suturing at four weeks under local infiltration anesthesia (4 mL Lignocaine 2% plus 7 mL Bupivacaine 0.5%) and moderate sedation using Ketamine and Propofol titrated dose under ASA standard monitoring. The procedure and postoperative period were uneventful. The steroid was weaned off on an outpatient basis and followed up until the next five months; she continues to do well.

## Discussion

Leprosy is a systemic disease involving multiple organ systems and requires MDT, which has significant side effects and impacts on the hepatic, renal, and hematological systems. The present case highlights the importance of thinking beyond the disease-related systemic derangement, drug therapy, and its side-effect management. Although leprosy is a slowly progressing disease, it has a crucial impact in the perioperative period. It can involve the respiratory system and airway, an indispensable organ system that anesthesiologists deal with. Atrophic changes in the nasal mucosa, impaired olfaction, loss of intranasal sensitivity, and laryngeal involvement with stridor, respiratory dysautonomia, altered pulmonary function parameters due to affliction of the vagus and sympathetic plexus were observed in leprous patients [3,4]. Altered baroreflex dysfunction and autonomic nervous function manifest as orthostatic hypotension and postprandial hypotension and can even lead to impaired myocardial contractility, hyper-reactive heart rate, and sudden cardiac death [5]. ST and T-wave changes, bundle branch block, extrasystoles, prolonged QT intervals, and arrhythmias are also noted [6]. Advanced cases might have hepatic and renal involvement, impairing metabolism and excretion of the drugs administered during anesthesia [7]. MDT used for the treatment also contributes to these hepatorenal effects. Skeletal deformities can be seen in digit resorption, chronic osteomyelitis, and osteoporosis in advanced cases [7].

The World Health Organization recommended MDT (Dapsone, Rifampicin, and Clofazimine) for the treatment of Leprosy, which exhibits mild to severe adverse drug reactions and can interfere with perioperative outcomes. Some critical side effects of Rifampicin are Cytochrome P450 activation, hepatotoxicity, drug-induced hepatitis, flu-like syndrome, thrombocytopenia, and acute renal failure [8,9]. Clofazimine-induced significant side effects are changes in skin color, rashes, itching, elevated blood sugar, retinopathy, nephrotoxicity, eosinophilic enteropathy, and cardiac arrhythmia [10]. Dapsone, a folate antagonist, has significant adverse effects, including hemolytic anemia, methemoglobinemia, agranulocytosis, abnormalities in liver function tests, jaundice, toxic or cholestatic hepatitis, peripheral neuropathy with primary motor function loss, psychosis, headache, dizziness, nausea, vomiting, dapsone hypersensitivity syndrome (DHS) [5,11]. DHS is a rare but life-threatening side effect of the drug characterized by fever, skin rash, leukemoid reaction, eosinophilia, thrombocytosis, lymphadenopathy, hepatic, pulmonary, and other systemic manifestations and requires immediate action [12]. DHS can develop anytime within seven days to six months of dapsone administration. Treatment includes early withdrawal of Dapsone, symptomatic treatment, and systemic corticosteroids (oral Prednisolone 1 mg/kg/day or intravenous methylprednisolone in equivalent doses) [12,13]. Dapsone-induced hemolysis and anemia are more profound in Glucose-6-phosphate dehydrogenase deficiency [14]. All these adverse effects may lead to impaired coagulation, decreased oxygen-carrying capacity, increased risk of infection, increased risk of postoperative nausea and vomiting, impaired drug metabolism, and delayed recovery from anesthesia.

The anesthesiologists usually consider this leprosy-related organ dysfunction and perform careful airway examination to rule out vocal cord involvements and the need for airway management, including the increased risk of pulmonary aspiration, infection, difficulty in nasopharyngeal airway placement, intubation, and delayed post-anesthesia recovery. Exposure keratitis risk prevention and careful positioning are needed to avoid pathological fractures during anesthesia [7]. Drug-drug interaction and the possible effect of the treatment-related drug therapy and anesthetic agent are other critical aspects that are considered in the perioperative period. Some drug effects, like sedatives and neuromuscular block, can also be objectively assessed and tailored accordingly.

However, immunological aspects of the causative agent, mainly when the disease is caused, are no longer considered infectious, and their perioperative implications and preparations are frequently not given the same consideration as provided for the other concerns mentioned above. Immunological responses to *M. leprae* antigen represent two distinct forms that can complicate the course of infection: Lepra type-1 and type-2 reactions or ENL. Type-1 reactions are delayed-type hypersensitivity reactions more common in borderline leprosy and are characterized by acute inflammation of skin, nerves, or both. [15]. ENL are immune complex-mediated responses affecting different organ systems. Prednisolone, a potent immunosuppressant, is used to treat these reactions.

Over and above MDT, trauma and stress have been implicated as triggering factors for ENL. The pathophysiology of ENL is multifactorial, and the role of increased neutrophil and their degranulation, T-cells, immune complex, and cytokines, especially tumor necrosis factor (TNF) and interleukins are documented [16]. Anesthetic drugs and techniques have been implicated in the immune system [17]. General anesthesia has been mostly reported to be pro-inflammatory, and regional anesthesia skews toward anti-inflammatory. However, epidural and spinal anesthesia have been shown to have insignificant effects on TNFα [18,19]. Our patient developed ENL despite being conducted under CSE. The patient might have had inflammation and cytokine excess due to the development of a surgical site infection. Prednisolone is a firm anti-inflammatory and immunosuppressant effective against ENL [20]. The second surgery under local anesthesia and moderate sedation did not trigger ENL, as it was probably under the beneficial effect of Prednisolone. Nevertheless, steroids in the perioperative period can also predispose to infections, and patients need to be chosen based on the risk involved and the benefits expected. Tailoring the patient selection for steroids towards only “at-risk” ENL and not having a risk factor for severe infection like immunodeficiency state, sepsis, etc., will be a good clinical practice. 

## Conclusions

Perioperative management of leprosy patients is still challenging owing to the multisystemic involvement. The hospital course might be complicated even with immunological adverse events; type-2 reaction, i.e., ENL, is one of them. Muti-disciplinary management is required, and steroids can effectively treat ENL. Surgery is a stress, and anesthetic drugs have an impact on immunological systems, which might predispose a leprosy patient on MDT to ENL. However, data are scarce for both postoperative ENL and prevention. Although a single case cannot provide causation or association, the present case hints towards the probable preventive action of steroids on the occurrence of postoperative ENL. Nevertheless, steroids in the perioperative period can also predispose to infections, and patients need to be chosen based on the risk involved and the benefits expected. Tailoring the patient selection for steroids towards only “at-risk” ENL and not having a risk factor for severe infection like immunodeficiency state, sepsis, etc., will be a good clinical practice. 
